# Public Hygiene and Legal Medicine

**Published:** 1861-01

**Authors:** H. W. Jones

**Affiliations:** Chairman


THE CHICAGO
MEDICAL EXAMINER

VOL. II. JANUARY, 1861. No. 1

### ORIGINAL CONTRIBUTIONS.

### REPORT OF COMMITTEE ON PUBLIC HYGIENE AND LEGAL MEDICINE.

NOVEMBER, 1860.

*Mr. President, and Gentlemen of the Chicago Academy of Medical Science:*

In pursuance of the object for which this committee was appointed, efforts have been made to gather information from all quarters of the city, respecting its general health, and the character of the diseases which have manifested themselves in our midst during the past year.

The result of our investigation has been a renewed conviction that Chicago has for this period, enjoyed a remarkable exemption from those severe and wide-spreading epidemics so frequently the scourge of both city and country in other parts of our land; a conviction hardly to be arrived at by a merely casual observer of our topography, unacquainted with the facts at hand.

The Fevers, both periodical and continued, which are always met with to greater or less extent, have been infrequent and mild, and the more important phlegmasiæ, Dysentary and Erysipelas, have but borne their due proportion in prevalence and degree.

In the summer months, the bowel-complaints of children were the chief claimants of our attention ; nor did these present any unusual features or require extended treatment. Fatal results were generally the consequence of long delay in applying for professional advice, occurring more commonly in children of the poorer classes, and those subjected to unfavorable hygienic influences.

Measles, Scarlatina and Hooping Cough, have not prevailed to any great extent, and for the most part have been mild in course, though in summer some cases were complicated with Diarrhœa, and a few in the third or fourth week, with a cough of great severity, took on cerebral symptoms, terminating fatally.

Cases of Pleurisy and Pneumonia have been rare and quite amenable to treatment.

The disease which has attracted most attention during the year past, has been Diphtheria. We quote the following from the experience of a well known practitioner: He says, "during the month of October, November and December 1859, I met with it most frequently on the extreme western and south-western border of the city. In the latter part of the winter and early spring, it seemed more prevalent in the north-western part of the city. During the summer months, I rarely met with a case anywhere.

In September, I again saw five cases in the north-western section, between west Kinzie and Third Streets, and during October, I have had six well marked cases on the eastern and southern border. In the autumn, and early winter of 1859, my cases presented symptoms of an active sthenic character, with a decided tendency to invade the Larynx, adding the peculiar symptoms of Croup. Primary attacks of Croup were common at that time."

This gentleman adds that the tendency just referred to, does not seem to him to characterize the disease as he has met with it during the month of September and October, 1860; that, "it seems to be accompanied by a lower grade of fever, an earlier prostration with earlier and more abundant secretion of mucous in the fauces and nostrils, emitting a decidedly putrid odor," and finally, notices "a more distinctly remitting type in the accompanying fever."

In general, we do not find it the opinion of physicians that Diphtheria has, during the year in question, at any time been entitled to be styled an epidemic. We may here notice a remarkable variety of opinion as to the pathognomonic symptoms of this disease, some practitioners making much more rigid discrimination between Diphtheria, Inflammatory Croup and Tonsillitis with ulcerated sore throat, than do others, who seem to regard all pseudo-membranous formations in the throat, as Diphtheritic, alike in kind and origin, but varying in degree.

As to the prevailing methods of treatment in this affection, we merely say that these vary quite as widely as do opinions in its diagnosis, though at present a general sustaining course seems to be agreed upon.

In the department of Legal Medicine, your Committee deem it advisable to call the attention of the Academy solely to the acknowledged fact, that criminal abortion, with all its attendant moral and physical evils, is becoming more and more frequent in our community.

It is, therefore, with a view to the enlightenment of the public mind upon this subject, and with the conviction that the immediate tendency of the practice is toward absolute Hindooism, that we urge upon the profession the adoption of some decided measures for the arrest of the spread and increasing frequency of this unnatural crime.

In conclusion, we offer some statistics, based upon the last census, which may prove interesting.

The ratio of mortality for the entire city of Chicago, for the year commencing Oct. 1st, 1858 and ending Oct. 1st, 1859, was as 1 to $60\frac{1}{4}$; and from the last date to Oct. 1st, 1860, 1 to to $55\frac{2}{3}$.

During these two years, the proportion of deaths of children under 5 years of age, was about 5-9ths of the whole mortality.

Mortality of South Division, (2 years), as 1 to $54\frac{1}{3}$

" " North " " " 1 to $64\frac{8}{9}$

" " West " " " 1 to $59\frac{2}{3}$

Respectfully submitted,

H. W. Jones, *Chairman.*

